# Simulation before fabrication: a case study on the utilization of simulators for the design of droplet microfluidic networks

**DOI:** 10.1039/c8ra05531a

**Published:** 2018-10-10

**Authors:** Andreas Grimmer, Xiaoming Chen, Medina Hamidović, Werner Haselmayr, Carolyn L. Ren, Robert Wille

**Affiliations:** Institute for Integrated Circuits, Johannes Kepler University Linz 4040 Linz Austria andreas.grimmer@jku.at robert.wille@jku.at; Department of Mechanical and Mechatronics Engineering, University of Waterloo 200 University Ave W. Waterloo ON Canada; Institute for Communications Engineering and RF-Systems, Johannes Kepler University Linz 4040 Linz Austria

## Abstract

The functional performance of passively operated droplet microfluidics is sensitive with respect to the dimensions of the channel network, the fabrication precision as well as the applied pressure because the entire network is coupled together. Especially, the local and global hydrodynamic resistance changes caused by droplets make the task to develop a robust microfluidic design challenging as plenty of interdependencies which all affect the intended behavior have to be considered by the designer. After the design, its functionality is usually validated by fabricating a prototype and testing it with physical experiments. In case that the functionality is not implemented as desired, the designer has to go back, revise the design, and repeat the fabrication as well as experiments. This current design process based on multiple iterations of refining and testing the design produces high costs (financially as well as in terms of time). In this work, we show how a significant amount of those costs can be avoided when applying simulation before fabrication. To this end, we demonstrate how simulations on the 1D circuit analysis model can help in the design process by means of a case study. Therefore, we compare the design process with and without using simulation. As a case study, we use a microfluidic network which is capable of trapping and merging droplets with different content on demand. The case study demonstrates how simulation can help to validate the derived design by considering all local and global hydrodynamic resistance changes. Moreover, the simulations even allow further exploration of different designs which have not been considered before due to the high costs.

## Abstract 2

Simulating microfluidic networks allows to check a design even before first prototypes are realized.

## Graphical abstract

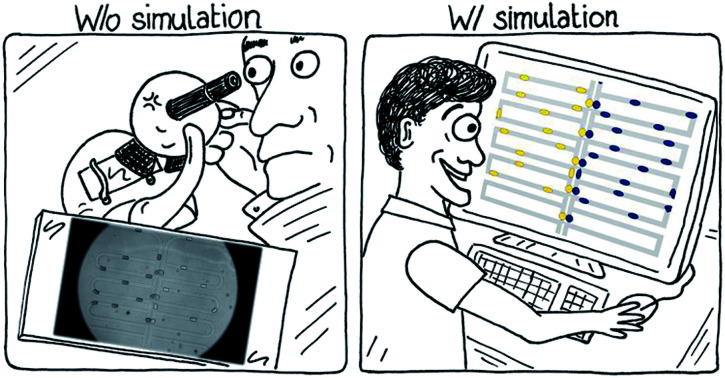

## 1 Introduction

There have been numerous studies reported on droplet microfluidics over the past decade as summarized by a number of review articles.^1–5^ Fundamental studies mainly focus on investigating and elucidating two phase flow and transport phenomena as well as exploring functionalities for droplet manipulation,^6–12^ while application driven studies aim to exploit droplet microfluidics to address the challenges associated with the current best practices.^13–18^ Both active and passive methods have been developed for manipulating droplets such as droplet generation, merging, splitting and trapping. Passive methods rely on the variation of applied pressures, geometries and fluid properties to manipulate droplets and thus do not need external components to be integrated with microfluidic devices. In general, passive methods are more favorable for large channel networks (*i.e.* large microfluidic networks) where integrating multiple active components becomes challenging.

However, the functional performance of passive methods is highly dependent on the channel dimensions and their fabrication precision as well as the applied pressures because the entire microfluidic network is coupled together meaning that any event (*i.e.* droplet generation, merging, splitting, or exiting of the channel network) occurring in the microfluidic network would change the local and global hydrodynamic resistance and thus the pressure drop over different channel sections. Furthermore, design and operational uncertainties such as fluctuations in the applied pressures, fabrication defects and dusts or particles entering the microfluidic network are inevitable in microfluidic studies.

Therefore, the design of the microfluidic network needs to be smart enough to be insensitive to these uncertainties. In other words, the designer has to derive the dimensions of the channel network, the applied pressures, *etc.* so that the resulting specification is as robust as possible. However, in this task, designers rely on their expert knowledge and derive the specification based on simplifications as well as assumptions. For example, designers often simplify or ignore time-dependent effects on the hydrodynamic resistance of microchannels caused by droplets because it is simply impossible to consider all droplet states and positions by hand.

In order to address these challenges, researchers such as Glawdel *et al.*^19^ presented a series of global network design criteria which are supposed to aid designers in determining the correspondingly needed specification. But, while certainly helpful, these criteria only narrow down the microchannel dimensions to a certain range. Using them, still multiple designs are required by varying the channel dimensions *i.e.* width, length and height within the range set by the network design criteria to figure out the optimal design. Furthermore, recently also an automatic method was presented in ref. 20, which aids the designer in the dimensioning task.

In order to validate the respective designs (which can reach up to dozens depending on the complexity of the microfluidic chip), usually physical experiments are conducted. In case that the functionality is not implemented as desired, the designer has to go back, revise the specification, and repeat the prototyping before he/she can conclude whether the revisions eventually lead to the desired result.

This design process apparently comes with major drawbacks: expensive physical experiments are used in order to validate designs. This often results in multiple iterations for prototyping where each iteration requires the generation of a physical design and the execution of the experiments – resulting in high costs (financially as well as in terms of time). Moreover, these circumstances frequently prevent the exploration of better and more advanced realizations (*i.e.* only several values can be picked in order to reduce the total number of prototypes).

In contrast, simulations can significantly help validate the design before the first prototype is fabricated. In fact, several simulation approaches have been introduced for simulating microfluidic designs in the past. These simulation approaches can be classified in two abstraction levels:

• Simulations using Computational Fluid Dynamics (CFD): in CFD-simulations, the fluid flow is described in the most detailed and accurate way which requires a complex simulation setup (*e.g.* the generation of a mesh based on the physical design). The high level of physical details causes high computational costs, which makes this kind of simulation most useful for (and also limits it to) small designs or parts of a larger design (*e.g.* single components). Reviews of corresponding methods are provided in ref. 21–23.

• Simulations using the one-dimensional (1D) circuit analysis model: this model reduces the microfluidic network which is inherently a 3D object to an 1D hydraulic circuit, which has an analogy to an electric circuit. This model can be applied when the flow is laminar, viscous, and incompressible.^24^ Furthermore, this model also considers the influence of droplets on the hydrodynamic resistance of microchannels.^25^ But it does not allow designers to predict effects like droplet mixing, formation or splitting.^25,26^ The precision of simulations on this level has been systematically evaluated by using physical experiments in *e.g.*ref. 25–31. Corresponding simulators are especially suited to simulate large microfluidic networks, even before starting with physical design.

For the purpose considered here, simulations on the 1D circuit analysis model are most suitable because they can be applied for large microfluidic networks and are perfectly applicable for design exploration. This makes them especially suited for addressing the previously discussed challenges: simulations allow users to validate the derived specification by considering all before applied simplifications and assumptions. Further, these simulations can be conducted at the beginning of the design process even before a physical design is drawn. If a simulation predicts that the chosen specification is not robust or even invalid, the probability is high that a prototype based on this specification will also show an incorrect functionality – which eventually reduces the number of tested prototypes. Furthermore, simulations allow the exploration of different designs.

But the potential of simulations on the 1D circuit analysis model has hardly been exploited by designers yet. In this work, we show the potential simulators can provide during the design of microfluidic networks. To this end, we consider the design of a microfluidic network – both, in the “traditional” fashion (*i.e.* manually, with many prototyping iterations) and in a fashion where a simulation is additionally used. As a case study, we use the microfluidic network presented in ref. 32, which consists of pairs of trapping wells. The working principle of the trapping well design is based on the hydrodynamic bypass concept, which has been reported by Takeuchi's group^33^ and Vanapalli's group^34^ for trapping single droplets. In ref. 32, those concepts have been improved for trapping pairs of droplets. The design does not require any electrodes, magnets, or any other moving parts and is entirely passively controlled by pressure and flow changes. In this study we show that, compared to the original design process which required six fabricated prototypes, one person month of an experienced designer, and financial costs of USD 1200, using simulation can significantly help when deriving the specification. More precisely, this study demonstrates that when using simulation, the designer is guided towards the design, which has been finally used in ref. 32. Moreover, the simulations even allow further exploration of new designs and *e.g.* prediction of their throughputs.

In the following, the applied 1D circuit analysis model is reviewed as well as the details of the considered microfluidic network and its implementing application are provided.

## 2 Background

In this section, we first review physical basics of microfluidic networks. Afterwards, we describe the considered case study and its application and working principle of the design.

### 2.1 Applied model

The design of microfluidic networks is usually conducted on the one-dimensional (1D) circuit analysis model, whose duality to electric circuit is reviewed in ref. 24. In this model, droplets can be considered as described in ref. 25. In the following, we briefly review this model.

For microfluidic systems at low Reynolds numbers, the relation between two points in a channel can be described by the Hagen–Poiseuille's law^35^ with

1 Δ*P* = *QR*,

where *Q* is the volumetric flow rate *Q* (in [μl min^−1^]), Δ*P* is the pressure gradient (in [mbar]), and *R* is the fluidic resistance (in [mbar min min^−1^]). This resistance of channels is constant for low Reynolds numbers and depends on the channel's geometry and the fluid viscosity *μ*_c_ (in [mPa s] when given as dynamic viscosity) of the continuous phase. For example, the resistance of a rectangular channel (with length *L*, width *w* and height *h*), where the section ratio *h*/*w* is less than 1, is given by2
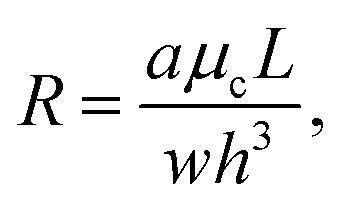
where *a* denotes a dimensionless parameter defined as3
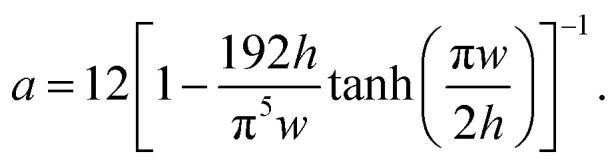


However, the presence of droplets in channels change the pressures and flow rates in the microfluidic network (*i.e.* the flow state) as they cause additional resistances. When the distance between two adjacent droplets is at least a few channel sections/diameters, their flow perturbations do not interact,^25^ which allows the modeling of each droplet by an additional resistance. The overall flow resistance of a microchannel can be calculated by

4 R* = *R* + *nR*_d_,

where *R* is the resistance of the channel, *n* is the number of droplets inside the microchannel, and *R*_d_ is the single droplet resistance.

The droplet resistance *R*_d_ is dependent on the capillary number Ca, the viscosity ratio of the dispersed phase to the continuous phase 
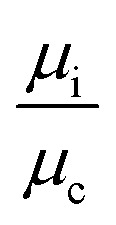
, the interfacial tension *γ* and also channel dimensions, which is described by^36^5
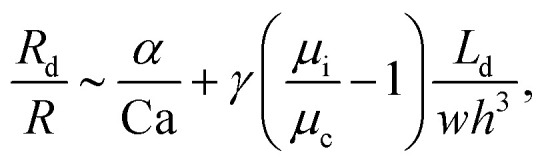
where *α* is a constant and *L*_d_ is the droplet length. In our case study, Ca and 
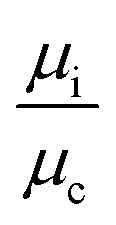
 are small 
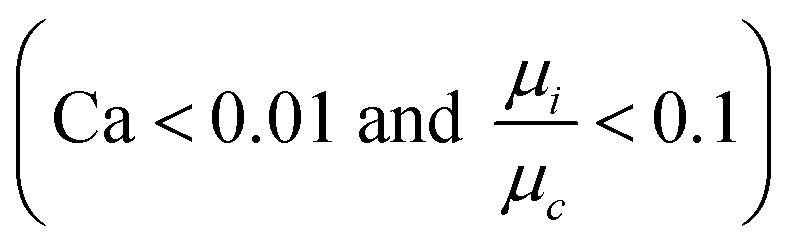
 and, hence, the viscosity of droplets can be neglected. Therefore, in this case study we apply the estimation for droplet resistances proposed in ref. 19 for simplicity. This estimation describes the droplet resistance by^19^6
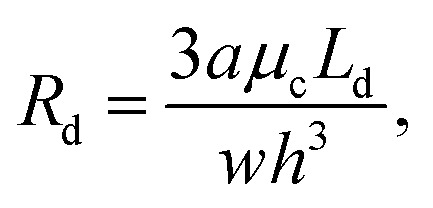
which has been experimentally verified. Furthermore, this estimation of the droplet resistance is only valid for a confined flow (*i.e.* where droplet lengths are greater than the channel width). For an unconfined flow (*i.e.* where the diameter of spherical droplets is smaller than the channel width), this equation needs to be changed.

In order to determine the flow state in all channels, the mass conservation and the relation described by the Hagen–Poiseuille equation can be employed.^25^ In detail, equations similar to the Kirchhoff's laws can be obtained by the following rules:

• The sum of flow rates into a node is equal to the sum of flow rates out of that node. A node is a point in the microfluidic network where the flow splits or merges.

• The directed sum of pressure gradients around any closed cycle is zero. The sign of the pressure gradients is defined by the specified direction of the flow rates.

By solving the resulting equation system, the flow state (*i.e.* Δ*P* and *Q*) in every channel for the current droplet positions can be determined. Note that this flow state becomes invalid as soon as a new droplet is injected, any droplet exits the network, or any droplet enters another channel (as this modifies the resistances in the equation system).

The obtained flow rates in the channels allow the determination of the droplet speed by7
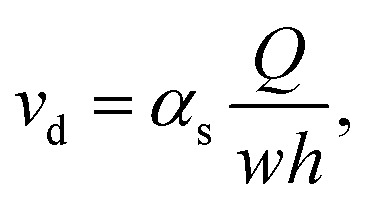
where *α*_s_ is the slip factor. Under the conditions where the droplet length is between 1.5*w* and 7.2*w*, the viscosity ratios is 0.03 or 0.88, and the capillary number between 0.001 and 0.01 without surfactant, Vanapalli *et al.*^37^ found the slip factor to be constant and equal to *α*_s_ = 1.28.

By using the obtained flow state, also pressure gradients can be checked whether or not they exceed the Laplace pressure, which *e.g.* would cause a droplet to be squeezed through a gap. The Laplace pressure is defined by8
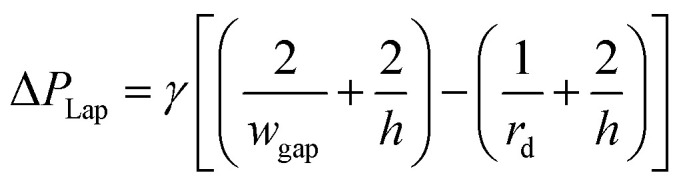
where *γ* is the interfacial tension (in [N m^−1^]), *w*_gap_ is the width of the gap, and *r*_d_ is the droplet radius.

### 2.2 Considered case study

In this case study, we consider a droplet microfluidic system, which can be used to screen drug compounds that inhibit the tau-peptide aggregation.^32^ This phenomenon is related to neurodegenerative disorders such as Alzheimer's disease^38^ and here protein misfolding and aggregation are considered to play a significant role. Therefore, the screen process is to figure out the compounds that can inhibit protein aggregation. The use of droplets for this application allows the significant reduction of the sample consumption volume by a factor of 100 as well as of the reaction time from 2 h to several minutes compared to the traditional 96-well plates.

Due to the droplet's large surface to volume ratio and fast mixing properties, the protein aggregation process reaches plateau within only 30 s. Hence, the reagents such as protein, fluorescent dye, and inhibitor, cannot be premixed before droplets get trapped for real-time monitoring, *i.e.* the reaction needs to be triggered on demand.

**Fig. 1:**
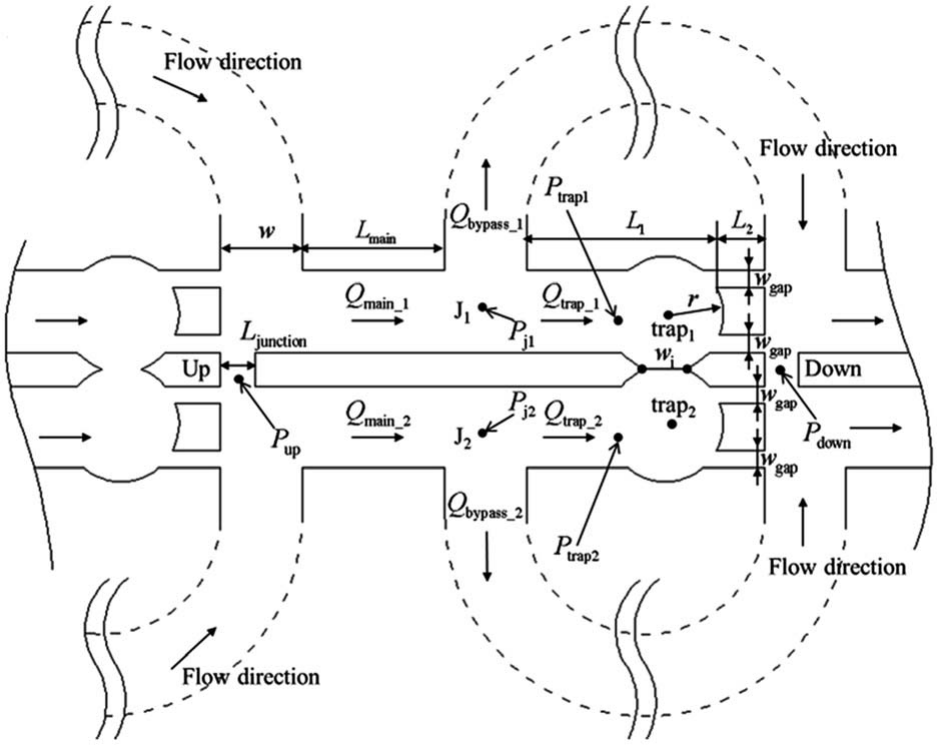
Trapping wells as proposed in ref. 32.† The shown trapping well pair allows to trap, merge, and mix droplets from two droplet streams. When a droplet reaches the entrance of a trapping well (*i.e.* point J_1_ or J_2_) and the respective trapping well does not yet contain a droplet, the droplet should flow into the trapping well. Therefore, the flow rate into the trapping well has to be larger than the flow rate into the bypass channel, *i.e. Q*_trap_ > *Q*_bypass_. Furthermore, a trapped droplet should stay in the trapping well and, hence, must not be squeezed into the other trapping well (*i.e. P*_trap_1__ − *P*_trap_2__ must not exceed the Laplace pressure) and must not be squeezed through the gaps downstream (*i.e. P*_trap_1__ − *P*_down_ must not exceed the Laplace pressure). On the other hand, when the trap already contains a droplet, following droplets should enter the bypass channel. Therefore, a trapped droplet has to decrease the flow rate into the trapping well so that the flow rate into the bypass channel gets larger, *i.e. Q*_trap_ > *Q*_bypass_.

†Published by The Royal Society of Chemistry.More precisely, when a droplet reaches the entrance of a trap and this trap does not already contain a droplet, the droplet has to enter the trap where the droplet should then stay (*i.e.* should not be pushed into the other trapping well or through any gap downstream). As soon as a droplet of the second reagent is trapped in the adjacent trap, both droplets merge and the reagents mix. On the other hand, when the trap already contains a droplet, following droplets should enter the bypass channel, which is again connected to further trapping wells. In addition to Fig. 1, the working principle of the trapping wells is also nicely illustrated by means of videos available at https://doi.org/10.1039/c7ra02336g.

This real-time monitoring functionality and the therefore required operations are implemented as a passive solution (*i.e.* no active components are required) in ref. 32. For producing droplets of the two reagents, the design needs two independent droplet generators (*i.e.* two T-junctions). The key elements of the design are trapping wells (also shortly called traps in the following) as shown in Fig. 1. Each set of trapping wells allows two droplets be trapped, merged, and mixed and, hence, allows the precise control of the reaction time.

In order to implement this desired behavior, two objectives have to be fulfilled:

(1) A droplet has to enter an empty trap: as a droplet always flows along the branch with the highest volumetric flow rate, the flow into the trap has to be larger than the flow into the bypass channel when the trap does not already contain a droplet, *i.e. Q*_trap_ > *Q*_bypass_. On the other hand, when the trap already contains a droplet, succeeding droplets in the stream have to enter the bypass channel. This is guaranteed by drastically decreasing the flow into the trap when the trapped droplet clogs the two narrow gaps, *i.e.* by ensuring *Q*_trap_ < *Q*_bypass_.

(2) A trapped droplet has to stay in the trap and must not be squeezed through any gap: first, to prevent the trapped droplet from being squeezed through the gap between the traps, the pressure drop between two connected traps must be less than the Laplace pressure across their intersection. Fig. 1 shows the intersection between both traps and its width *w*_i_. Furthermore, when the trap contains a droplet, the droplet's radius is *r*_d_ ≤ *r*. This allows the definition of the objective as^32^9
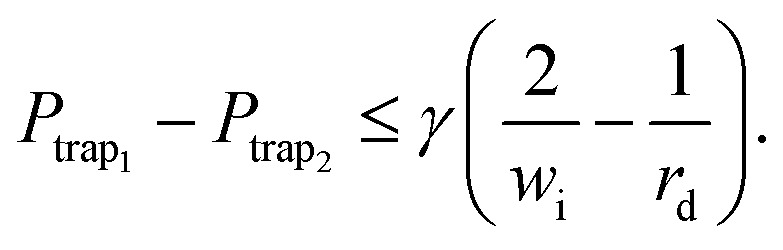


Second, to prevent a trapped droplet from being pushed out of the trap, a similar objective has to be fulfilled,^32^*i.e.*10
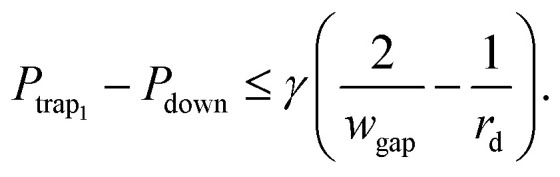


In this work, we aim to discuss the design of this non-trivial microfluidic network and how this can be advanced using simulation. Therefore, we next review the current process of designing microfluidic networks.

## 3 Current design process

In this section, we review and discuss the steps and respective challenges in the current process of designing a microfluidic network. Therefore, we use the case study as introduced in the previous section.

### 3.1 Deriving the specification

Taking the desired behavior and the basic structure of the design (*cf.*Fig. 1), the dimensions of all channels, the applied pressures/flow rates, and the used phases have to be specified next, *i.e.* the designer has to derive a specification.

Here, some dimensions are given by the application or by fabrication limitations, *e.g.*:

• The droplets' size and spacing determine the trapping well radius *r* and the length *L*_1_, *i.e. r* is chosen so that a droplet fills most of the trap and *L*_1_ is chosen so that the following droplets do not contact with the trapped droplet.

• The fabrication limits the gap width *w*_gap_ and gap length *L*_2_ (*i.e.* the pillars require a minimum size, otherwise they could be peeled off from the silicon wafer).

• The properties of the used phases are chosen so that they are suited for forming droplets and for the application.

However, besides that, all remaining issues such as the length of the bypass channel or the applied pressures have to be explicitly specified by the designer. Considering that even small droplet-based microfluidic networks are composed of several channels which, together with the droplets themselves, yield to plenty of interdependencies, thus this leaves a tedious task. In fact, the authors of ref. 32 spent a significant portion of their work discussing the respective issues in detail, which is why we refer to this work for a more detailed treatment.

Overall, deriving the specification requires the definition and consideration of plenty of variables which all affect the intended behavior (*i.e.* whether the objectives are fulfilled). In this task of deriving the specification, the designer relies on his/her expert knowledge and often applies simplifications and assumptions. Especially, the time-dependent resistances caused by droplets are often simplified or completely ignored when deriving the specification as it is impossible to consider all droplet states and positions by hand. For example, in the specification of ref. 32, only for the bypass channel a fix number of contained droplets is assumed, which causes an additional resistance.

### 3.2 Prototyping and testing

The task reviewed in the previous section eventually yields a specification which is supposed to realize the intended behavior. However, due to applied simplifications and assumptions, the designer cannot be sure whether an implementation based on the specification indeed realizes the desired behavior and whether all objectives are fulfilled under all settings.

Therefore, as a next step, it is tested whether the specification realizes the desired functionality by using (physical) experiments. To this end, the designer has to fabricate the design based on the derived specification. This first requires a physical design (also called layout or mask) to be drawn from the specification (*e.g.* as a vector graphic), which can be used as input for the production process. Afterwards, this is used to fabricate the device using a soft-lithography technique, 3D-printing, or milling. The resulted device then is validated, *i.e.* experiments are conducted to check whether the device indeed shows the desired behavior. This is the first time in the design process in which the designer can observe the effects of his/her choices and decisions as well as simplifications and assumptions during the derivation of the specification (which provided the basis for the physical realization) on the actual behavior.

In the case that those validations show that the behavior has not been implemented as desired, the specification has to be refined, *i.e.* the dimensions of some channels, the applied pressure/flow rates, or some of the used phases have to be adjusted. In particular at the beginning of the design process, this of course is likely needed as assumptions might be inappropriate and simplifications may have led to an imprecise specification. But then, the entire process of creating a physical design and fabricating another prototypical device has to be repeated in order to test again whether the (now refined) design is correct. This iteration of refining and testing constitutes one of the major drawbacks of today's design process as it requires a significant amount of time and resources.

In fact, in the case study conducted in ref. 32, a total of six different specifications had to be derived, fabricated, and tested until the desired behavior was accomplished. More precisely, prototypes with bypass channel lengths and trapping well gap widths as shown in Table 1 have been fabricated.

**Table 1:** Tested bypass channel lengths and trapping gap widths of the fabricated prototypes

ID	*L*_bypass_	*w*_gap_
1	3000 μm	15 μm
2	4000 μm	15 μm
3	5000 μm	15 μm
4	3000 μm	25 μm
5	4000 μm	25 μm
6	5000 μm	25 μm

Each of these prototypes has experimentally been tested for the trapping efficiency, and the one with ID 2 (*i.e.* a bypass channel length of 4000 μm and trapping well gap width of 15 μm) eventually showed the desired performance with respect to trapping robustness.

Overall, this resulted in a working time of one month for an experienced designer and financial costs (including silicon wafer, SU-8 photo resistor, fee charged for clean room, Polydimethylsiloxane (PDMS), and silicone oil, *etc.*) of approximately USD 200 for a single prototype (*i.e.* a total of USD 1200 until the desired design eventually worked).

### 3.3 Further missed potential

The complexity of the design process as reviewed above does not only pose a challenge to get a design realizing the desired behavior. Moreover, it also prevents further improvements which, in principle, could be conducted but are too expensive in most cases. Hence, as soon as a prototype shows a correct behavior, the respective design and its specification is usually fixed and no more different designs are explored. However, probably different designs would be even more suited, *e.g.* would be more robust, would be smaller, would increase the throughput or would have a positive effect on the application.

For the case study, no design has been explored to answer the following questions:

(1) Question 1: What is the minimal bypass channel length so that droplets still get trapped?

(2) Question 2: What is the maximum pressure over five sets of trapping wells so that no objective is violated?

(3) Question 3: How many trapping wells can be cascaded and loaded by droplets in a given time, *i.e.* what is the maximal throughput?

Why these questions are important and how to address them will be discussed later in Section 4.2.2.

Overall, it can clearly be seen that the design process as conducted thus far is certainly not ideal. Deriving a working specification requires several iterations of physical design and prototyping which becomes a time-demanding and costly process. Moreover, as a consequence, often the first working design is eventually used even if better and more advanced solutions would, in principle, be possible.

## 4 Improved design process using simulation

In this work, we aim for improving the design process reviewed above by using numerical simulations based on the 1D circuit analysis model. These simulations allow the iterations without the need for a physical design nor the testing on an actually fabricated device. How this is accomplished is covered in this section. To this end, we first review the used simulator as well as its features and application. Afterwards, we revisit the issues discussed in the previous section and describe how simulation can help to addressed them in an efficient way.

### 4.1 Used simulator

In this section, we describe the used simulator, which is applicable for simulating droplet microfluidics operated in a laminar, viscous, and incompressible flow regime. The descriptions are provided in two parts: first, we review its general working principle, which is supposed to provide a background on the internals of the simulator but can easily be omitted by readers who are only interested in a user-perspective of the simulator. Afterwards, we summarize the interface and core features which result from this working principle and, hence, are available to designers.

#### 4.1.1 Internal working principle

In this work, we employ the simulator presented in ref. 39, which has been implemented in Java (which makes the tool platform-independent) and can be downloaded at http://iic.jku.at/eda/research/microfluidics_simulation.

The simulator automatically derives, applies, and solves microfluidic equations based on the model described in Section 2.1. Therefore, the microfluidic network is represented as a directed graph. The edges in the graph represent channels and pumps and their directions represent the counting direction of the flow rates. The nodes represent points in the microfluidic network where edges are connected. This directed graph is used to automatically derive an equation system by applying the Kirchhoff's laws as described in Section 2.1. More precisely, the algorithm generates an equation for each node (*i.e.* in each node the sum of all incoming flow rates is equal to the sum of all outgoing flow rates) as well as for each cycle (*i.e.* the sum of all pressure gradients around a closed cycle is equal zero). For determining the cycles in the graph, a variant of Paton's algorithm^40^ is used.

In this equation system, also the effects of droplets are considered. However, the flow state becomes invalid as soon as a new droplet enters a channel/exits the network. Consequently, the entire equation system has to be frequently re-evaluated – which would be impossible by hand. The simulator conducts this automatically and solves the equation system by applying a lower-upper (LU) decomposition.^41^

Overall, the simulation algorithm performs the following steps in a loop:

(1) Compute flow state: the simulator computes the flow state (*i.e.* the pressures and flow rates in all channels) by considering all droplets and their positions. Therefore, the simulator automatically derives an equation system which considers the resistances caused by all droplets and solves it. The resulted flow rates allow the determination of the droplet speeds, *i.e.* by eqn (7).

(2) Check objectives: based on the obtained flow state, the objectives (*e.g.* a pressure must not exceed the Laplace pressure) are checked and in case of any violation, the simulator informs the designer.

(3) Compute next event-time: the next event time is computed by the minimum time until a new droplet is injected or any droplet enters the next channel/exits the network (here the droplets' speeds are used).

(4) Update system state: finally, the simulator updates the system state to this event time (*i.e.* the droplet positions and their resistances). For the droplet causing the event, the flow state decides which channel this droplet enters next (*i.e.* the channel with the highest flow rate).

Summarizing, the simulator re-calculates the flow state when the old one becomes invalid. This allows the simulator to consider all interdependencies caused by droplets. Furthermore, these event-based calculations make the algorithm efficient. More precisely, computing the flow state can be done in polynomial time with respect to the number of variables in the equation system and checking the objectives, computing the next event-time, as well as updating the system states is computationally inexpensive. This algorithmic efficiency allows to simulate large microfluidic networks in negligible computation times.

#### 4.1.2 User interface and features

The designer can use the simulator as a black box and, as already stated, he/she does not need to understand the algorithm in detail. Further, he/she does not need to provide a physical design yet as all simulations are conducted based on the information which is available to the designer anyway when he/she has completed the specification. More precisely, for setting up a simulation, the designer only has to provide the specification which describes

• the channel dimensions,

• the structure of the network (*i.e.* how are the channels connected),

• the applied pressures or volumetric flow rates from the inlets,

• the properties of fluids (*i.e.* the viscosities, densities, interfacial-tension), and

• the objectives which have to be fulfilled.

After providing the specification, the simulator allows the analysis of

• the instantaneous flow state of all channels in the microfluidic network,

• the trajectories of droplets (*i.e.* the droplet switching at bifurcations or whether they enter a trapping well) and their spacing and patterns,

• the droplet speeds and the residence time (*i.e.* the time for passing a channel), as well as

• the objectives whether they are fulfilled.

**Fig. 2:**
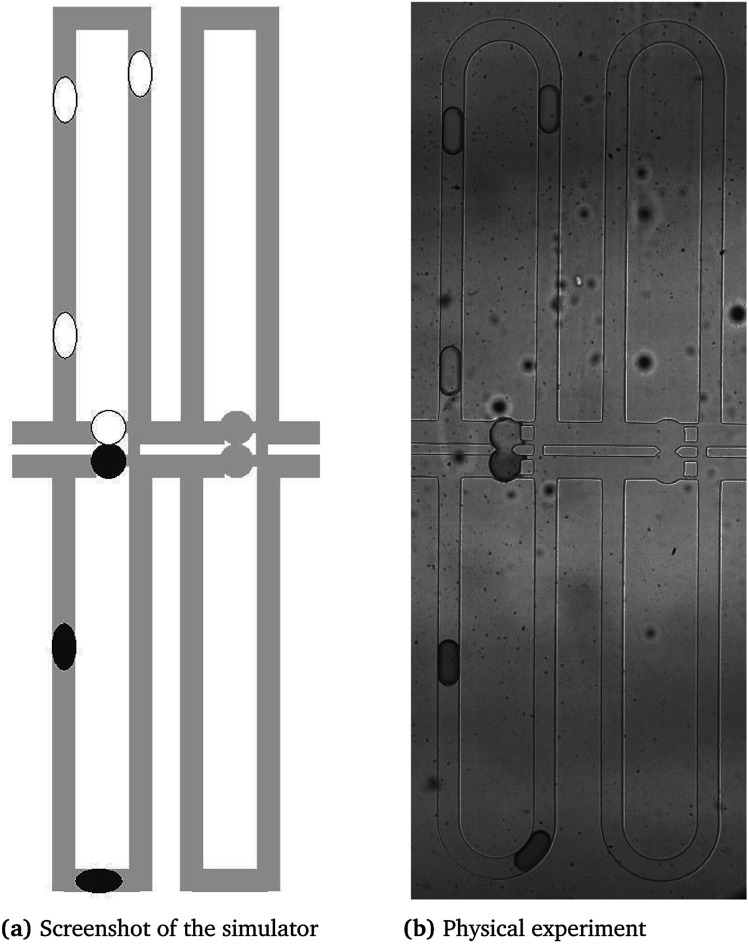
Comparison of the output of the simulator with the physical experiment.

The simulator's features can be observed in the video at http://iic.jku.at/eda/research/microfluidics_simulation, which shows the graphical output when simulating the chip of ref. 32 (*cf.* also considered in our case study). Additionally, Fig. 2 provides a comparison of the simulator and the physical experiment. On the left side of this video and figure, the graphical output of the simulation is shown which shows the droplet positions. On the right side of this video and figure, the simulation is compared to a physical experiment. Therefore, we used images of a physical experiment captured with a frequency of 50 fps. When comparing the simulation with the experiment, we can observe equal droplet trajectories as well as similar droplet speeds and residence times. Furthermore, the simulator constantly checks the objectives introduced in Section 2.2. The obtained results prove the precision with respect to a physical implementation.

### 4.2 Application of the simulator

The simulator and its features as reviewed above can now be utilized in order to address major shortcomings of the current design process for microfluidic networks as reviewed in Section 3. Recall that the designer derives the specification based on manual calculations, simplifications, as well as assumptions and can, thus far, only validate the specification by fabricating and testing the resulted prototype. Utilizing the simulator, many of these tests can now be conducted much earlier in the design process and without the need for either a physical design or a fabricated chip. This additionally allows for a much more elaborated design exploration as variances of the specification can be validated much easier, with significantly less manpower, and basically no cost. In the following, this is demonstrated by (1) revisiting the design process reviewed in Section 3.1 as well as Section 3.2 with respect to the features provided by the simulator and (2) illustrating the further potential which can be gained by the improved possibilities with respect to design exploration (and, thus far, has been missed as discussed in Section 3.3).

#### 4.2.1 Utilizing the simulator in the design process

In ref. 32, the designer came up with six different specifications to be tested using physical experiments. Instead of drawing six physical designs, fabricating respective prototypes, and conducting the experiments, in the following we evaluate these six specifications by utilizing the simulator.

The simulation of the specification with bypass length *L*_bypass_ = 3000 μm and gap width *w*_gap_ = 15 μm predicts that the flow into an empty trap is equal to *Q*_trap_ = 2.054 μl min^−1^ and the flow into the bypass channel is equal to *Q*_bypass_ = 1.871 μl min^−1^. Due to the fact that a droplet always flows along the path with the highest flow rate, a droplet enters the empty trap under perfect conditions. However, the flow rate ratio does not allow a robust decision of the droplet path. For example, even a small particle blocking the flow into the trapping well would reverse the ratio and, therefore, would violate the Objective 1 (*Q*_trap_ > *Q*_bypass_, *cf.* Section 2.2).

Using simulations, we found that this flow rate ratio between the trapping well and the bypass channel increases when the bypass channel length is increased and/or the gap width is increased (which is the case for all other specifications). However, an increase of the bypass channel length also causes an increase of the time that a droplet requires to pass this bypass. For example, the simulation showed that, when increasing the bypass channel length from *L*_bypass_ = 4000 μm to *L*_bypass_ = 5000 μm (using a gap width of *w*_gap_ = 15 μm), the time that a droplet requires to pass a single bypass increases from 0.25 s to 0.32 s (which is an increase of 28%). This effect adds up when multiple trapping wells are cascaded. When additionally a certain upper limit for the loading-time of droplets (*i.e.* especially relevant for cells) has to be fulfilled, less trapping wells can be cascaded using a longer bypass channel, which decreases the throughput.

On the other hand, the time for droplets to be trapped can be reduced by increasing their speed, which can be achieved by applying higher pressures at the inlets. Using the simulator, we tested different pressures for all six specifications and the simulator predicts that the pressure is limited by the Objective 2 (*cf.* Section 2.2). More precisely, too high pressures at the inlets cause too high pressure gradients between the trapping wells and the narrow gaps so that the condition described in eqn (10) is violated. This causes the trapped droplet to be pushed through the two narrow gaps. The simulation shows that droplet speeds can be higher for the prototypes which have a gap width of only 15 μm. This is because a smaller gap width increases the Laplace pressure.‡‡Note that, further details on the maximal possible pressure are provided later in the next section discussing design exploration possibilities.

These simulation results now allow the designer to evaluate the robustness and performance of the different specifications. Table 2 summarizes the obtained insights. Based on these results, the designer can evaluate the specifications with respect to their robustness. The results show a clear preference for the specification with ID 2. Exactly this specification is the one which was eventually realized in ref. 32 (*cf.* Section 3.2). The appealing features of this simulator are clear: instead of six fabricated prototypes, 1 person month of manual labor, and a total of USD 1200 of further costs, utilizing the simulator would allow for obtaining the same result by only fabricating one single design, spending only 1/6 of the previously spent time of manual labor plus few hours for simulating, and a total of USD 200. Apparently, this is a significant improvement compared to the design process applied in ref. 32.

**Table 2:** Robustness evaluation

ID	*L*_bypass_	*w*_gap_	Possible problems
1	3000 μm	15 μm	No robust flow rate ratio (violation of Objective 1 possible)
2	4000 μm	15 μm	—
3	5000 μm	15 μm	Bypass length decreases throughput
4	3000 μm	25 μm	Sensitive to high input pressures (violation of Objective 2 possible)
5	4000 μm	25 μm	Sensitive to high input pressures (violation of Objective 2 possible)
6	5000 μm	25 μm	Sensitive to high input pressures (violation of Objective 2 possible), bypass length decreases throughput

#### 4.2.2 Utilizing the simulator for design exploration

As discussed in Section 3.3, the complexity of the design process is not only a burden to get a design realizing the desired behavior, but also prevents the designer from exploring even better solutions. Utilizing the simulator, this burden is significantly reduced. In fact, as described next, all questions raised in Section 3.3 can now efficiently be addressed.

##### Question 1: What is the minimal bypass channel length?

Here, we explore the design with respect to the limits of the bypass channel length. Recall, a short bypass channel length decreases the time for a droplet to be trapped and hence affects the throughput of the design. Furthermore, a short bypass channel length also minimizes the area of the physical design, which is an important criterion of the design due to the limited space of a typical microfluidic chip.

The bypass channel lengths determine the flow rate into the bypass *Q*_bypass_ and also into the trapping well *Q*_trap_. Therefore, the bypass channel length is limited by the Objective 1 (*cf.* Section 2.2), *i.e.* the flow rate into an empty trapping well has to be larger than the flow rate into the bypass channel.

In order to explore the limits, we conducted simulations where we stepwise reduce the bypass channel length in the specifications and stop as soon as the first droplet does not enter an empty trapping well (which violates the objective *Q*_trap_ > *Q*_bypass_).

For a gap width of *w*_gap_ = 15 μm and for a gap width of *w*_gap_ = 25 μm, the simulation predicts a minimal bypass channel length of *L*_bypass_ = 2800 μm and *L*_bypass_ = 900 μm, respectively. Both values are only theoretical limits and respective designs would be sensitive to any imperfections as *e.g.* dust or imprecisions caused by the fabrication process. Therefore, a prototype will never be pushed to these limits. Instead these limits allow the designer to estimate the robustness of the design. For example, this design exploration would have prevented a prototype with *L*_bypass_ = 3000 μm and *w*_gap_ = 15 μm (*i.e.* the specification with ID 1 from Table 2) to be considered in the first place.

##### Question 2: What is the maximum pressure over five sets of trapping wells so that no objective is violated?

By increasing the pressure at the inlets, also the droplet speed is increased and, accordingly, a higher droplet speed decreases the time required for a droplet to travel from its injection until it gets trapped. This might be crucial since, the droplet loading time is limited for some biological experiments and, therefore, the droplet speed is an important factor for the throughput of a design.

In this design exploration, we explore for the considered design with five trapping wells (as proposed in ref. 32) the maximal possible pressures across these trapping wells. Again, we increment the pressures until any objective is violated. The simulation results reveal that the Objective 2 limits the maximal pressures. More precisely, for too high pressures, the simulation predicts that the pressure across the trapping well and the narrow gaps exceeds the Laplace pressure, which would cause a droplet to be squeezed out of the trap.

Table 3 shows the obtained results for the six specifications proposed in Section 3.2. These results show that a smaller gap width allows for a higher pressure drop. This can be explained because the Laplace pressures is higher for smaller gaps. Interestingly, also the shorter the bypass channel, the higher the possible pressure. These results again confirm that a gap width of only 15 μm is more robust for higher pressures.

**Table 3:** Maximal pressure drops

*L*_bypass_	*w*_gap_	Maximal pressure drop over five traps
3000 μm	15 μm	169 mbar
4000 μm	15 μm	149 mbar
5000 μm	15 μm	135 mbar
3000 μm	25 μm	65 mbar
4000 μm	25 μm	57 mbar
5000 μm	25 μm	52 mbar

##### Question 3: How much trapping wells can be cascaded and loaded by droplets in a given time?

The design proposed in ref. 32 cascades five trapping wells. However, it would be possible to cascade more trapping wells in order to increase the throughput. This number of cascaded trapping wells determines the time until all droplets are trapped, *i.e.* the loading time. The maximal allowed loading time depends on the bio-assays and can particularly be relevant to cells. Furthermore, the pressure drop over the trapping wells must not exceed operating settings, *i.e.* exceeding pressures will bow the PDMS channels. Typically, the pressure applied to PDMS microfluidic devices is limited to 5 bar.

In the current design process, it would be costly to explore designs with different numbers of trapping wells and measure the required loading time. Therefore, it is currently unexplored how many trapping wells can be cascaded so that all droplets can be trapped within a certain maximal loading time. Utilizing the simulator, now also this question can be easily explored.

Therefore, we create specifications containing between 15 and 45 pairs of cascaded trapping wells. For each of these designs, three different pressures (100 mbar, 200 mbar, and 300 mbar) over the trapping wells are applied. These pressures and especially the maximal pressure have been selected so that no objective is violated. Then, the simulator is used to measure the time which is required for all droplets to be trapped, *i.e.* the overall loading time.

**Fig. 3:**
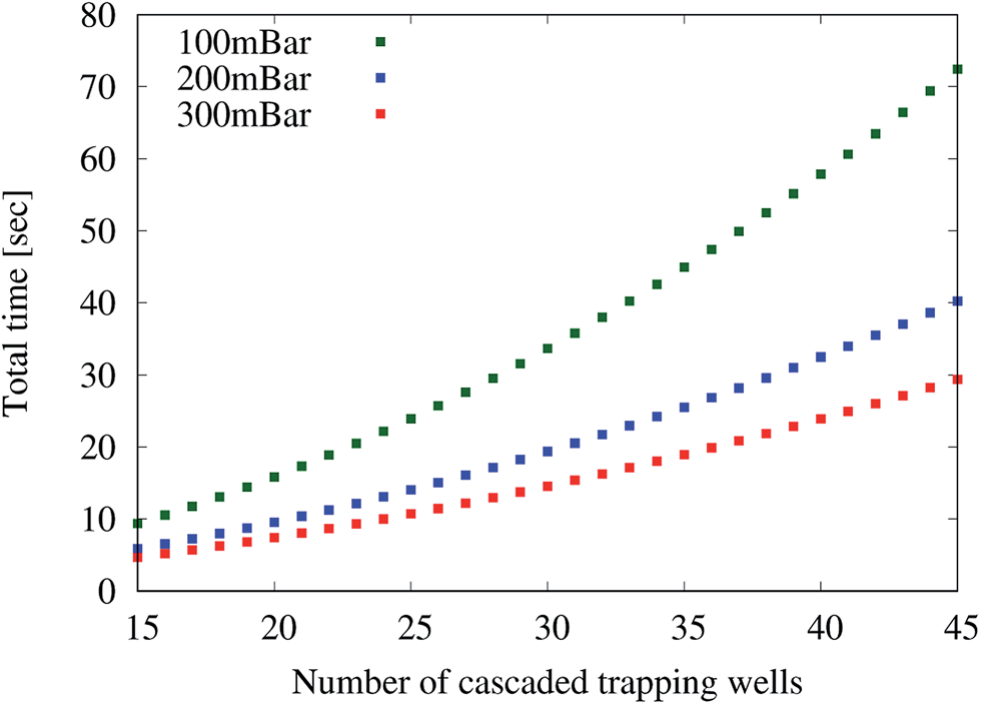
Throughput analysis.

Fig. 3 summarizes the obtained results for the three different pressures. We can see that the loading time increases with the number of cascaded trapping wells and is generally lower when higher pressures are applied. This increase can be explained because (1) the distance to the last set of trapping well increases and (2) the droplet speed decreases as the overall resistance caused by the trapping well increases. Overall, also here the simulator allows for obtaining results that were not available before – here, to implement a bio-assay with a maximal loading time and a maximal throughput.

## 5 Conclusion

In this study, we demonstrated how simulation can help in the design process of droplet microfluidics. Therefore, we conducted a case study comparing the design processes of the microfluidic design proposed in ref. 32 with and without simulation. When no simulation was used, the designer validated the specification by using physical experiments, which resulted in six prototypes, took one person month and produced financial costs of USD 1200. If the designer is not experienced, the costs could even be much higher. Instead, when a simulation was used (the used simulator is available at http://iic.jku.at/eda/research/microfluidics_simulation), the designer was able to validate the specification before any prototype and even before any physical design was made. These simulations allow the prediction of the robustness of the respective specifications and, in fact, show a clear preference for the specification which was eventually realized in ref. 32. Hence, the simulations allow for selecting the most robust design without the need to explicitly fabricate and test them. Furthermore, the simulations even allowed to explore further designs, which were too costly to consider in the current design process without simulation.

Overall, the use of simulation on the 1D circuit analysis is perfectly suited to address the wide variety of microfluidic networks and their respective design challenges. Simulations allow for a quick validation and exploration of arbitrary microfluidic designs (the setup of the simulation is hardly any work compared to physical experiments as well as the computation time sums up to at most a few seconds). Furthermore, they significantly help to increase the robustness of the design as well as to accelerate the design process and, by this, reduce the overall costs.

## Conflicts of interest

There are no conflicts to declare.

## Symbols

Δ*P*: Pressure gradient
Q
: Volumetric flow rate
R
: Resistance of a channel without droplets
L
: Channel length
W
: Channel width
H
: Channel height
*μ*_c_
: Fluid viscosity of carrier fluid
*μ*_i_
: Fluid viscosity of the droplet fluid
*R*_d_
: Resistance of a droplet
*L*_d_
: Droplet length
R*: Resistance of a microchannel with droplets
*v*_c_
: Speed of the carrier fluid
*v*_d_
: Speed of the droplet
*α*_s_
: Slip factor
Δ*P*_Lap_: Laplace pressure
Γ
: Interfacial tension
*r*_d_
: Droplet radius

## Supplementary Material
